# Has the STARD statement improved the quality of reporting of diagnostic accuracy studies published in *European Radiology*?

**DOI:** 10.1007/s00330-022-09008-7

**Published:** 2022-07-30

**Authors:** Ann-Christine Stahl, Anne-Sophie Tietz, Benjamin Kendziora, Marc Dewey

**Affiliations:** 1 Department of Radiology, Charité - Universitätsmedizin Berlin, joint Medical Faculty of Humboldt-Universität zu Berlin and Freie Universität Berlin, Berlin, Germany; 2 Department of Dermatology and Allergy, University Hospital, Ludwig Maximilian University, Munich, Germany

**Keywords:** Checklist, Accuracy, Diagnostic tests, Reference standards, Research design

## Abstract

**Objectives:**

To investigate whether encouraging authors to follow the Standards for Reporting Diagnostic Accuracy (STARD) guidelines improves the quality of reporting of diagnostic accuracy studies.

**Methods:**

In mid-2017, *European Radiology* started encouraging its authors to follow the STARD guidelines. Our MEDLINE search identified 114 diagnostic accuracy studies published in *European Radiology* in 2015 and 2019. The quality of reporting was evaluated by two independent reviewers using the revised STARD statement. Item 11 was excluded because a meaningful decision about adherence was not possible. Student’s *t* test for independent samples was used to analyze differences in the mean number of reported STARD items between studies published in 2015 and in 2019. In addition, we calculated differences related to the study design, data collection, and citation rate.

**Results:**

The mean total number of reported STARD items for all 114 diagnostic accuracy studies analyzed was 15.9 ± 2.6 (54.8%) of 29 items (range 9.5–22.5). The quality of reporting of diagnostic accuracy studies was significantly better in 2019 (mean ± standard deviation (SD), 16.3 ± 2.7) than in 2015 (mean ± SD, 15.1 ± 2.3; *p* < 0.02). No significant differences in the reported STARD items were identified in relation to study design (*p* = 0.13), data collection (*p* = 0.87), and citation rate (*p* = 0.09).

**Conclusion:**

The quality of reporting of diagnostic accuracy studies according to the STARD statement was moderate with a slight improvement since *European Radiology* started to recommend its authors to follow the STARD guidelines.

**Key Points:**

*• The quality of reporting of diagnostic accuracy studies was moderate with a mean total number of reported STARD items of 15.9 ± 2.6.*

*• The adherence to STARD was significantly better in 2019 than in 2015 (16.3 ± 2.7 vs. 15.1 ± 2.3; p = 0.016).*

*• No significant differences in the reported STARD items were identified in relation to study design (p = 0.13), data collection (p = 0.87), and citation rate (p = 0.09).*

**Supplementary Information:**

The online version contains supplementary material available at 10.1007/s00330-022-09008-7.

## Introduction

Studies of diagnostic accuracy compare the results of one or more tests under investigation with the results of the reference standard, which is the best available method for the detection of the target condition [1, 2]. Such studies tend to be prone to bias and variation, especially concerning demographic features, disease prevalence and severity, clinical review bias, and observer and instrument variation [3–8]. Biased results have an impact on the recommendations of the test under consideration and can hamper their generalizability [9, 10]. Because of this documented poor reporting quality in diagnostic accuracy studies, it is often difficult or impossible to judge the internal and external validity of a study [11]. To improve this situation, the Standards for Reporting Diagnostic Accuracy (STARD) statement was published in 2003 [12, 13]. STARD contains a checklist of 25 essential items which can help authors or reviewers to judge the introduction, methods, results, and discussion of a study more easily and detect potential sources of bias or variation [12]. A generic flow diagram for studies of diagnostic accuracy was also developed and shows included and excluded patients with reason, the number of participants at each stage of the study, and the distribution of the test results [12, 14]. In October 2015, the checklist was revised because of recent evidence about sources of bias, applicability concerns, and factors facilitating generous interpretation in test accuracy research [14]. Now there are 30 numbered items to judge diagnostic accuracy studies. Several of them are the same as in the original version published in 2003, others have been combined, split, or added [14]. Several studies have investigated whether the reporting quality of diagnostic accuracy studies in imaging journals has changed after the publication of the STARD statement [11, 15–19]. While the studies have identified an improvement, they also show that many authors still have to work on giving every information that is needed [11, 19–23]. Specifically, authors need to be more aware about the importance of reporting inclusion criteria and sampling methods for recruiting patients, information about blinding, and confidence intervals for accuracy estimates [11].

In mid-2017, *European Radiology* started encouraging its authors to follow the STARD statement in its submission guidelines. Although the use of the STARD guidelines is still not mandatory for authors, we conducted a study to investigate whether encouraging authors to follow the STARD guidelines has already improved the quality of reporting of diagnostic accuracy studies. We used the STARD checklist and analyzed 114 diagnostic accuracy studies published in *European Radiology* in 2015 and 2019.

## Materials and methods

This study was reported according to the PRISMA reporting guidelines for systematic reviews and meta-analyses [24], but was not registered in PROSPERO because it did not fulfill the inclusion criteria [25].

### Data sources

Two reviewers (A.S., an advanced medical student, and A.T., a dentist, both with 1 year of experience in performing literature reviews of diagnostic accuracy studies) independently searched MEDLINE (using PubMed) with a search strategy validated by Devillé et al to identify articles on diagnostic accuracy published in 2015 and 2019 as follows: “sensitivity AND specificity.sh” OR “specificity.tw” OR “false negative.tw” OR “accuracy.tw” (where “.sh” indicates MEDLINE subheading and “.tw” indicates text word) [20, 26]. The search was consequently limited to articles published in English and studies focusing on human subjects. For comparison, we chose the years 2015 and 2019 because we wanted to analyze studies published before the COVID-19 pandemic and because *European Radiology* started encouraging its authors to follow the STARD statement in mid-2017. This information was obtained from the editorial staff of *European Radiology* via e-mail. Only articles published in *European Radiology* were included in our investigation. MEDLINE was last searched on April 8, 2020. To verify that no relevant articles were missed, we additionally did a manual search of the *European Radiology* website for diagnostic accuracy studies published in 2015 and 2019. The last search was performed on June 23, 2020.

### Study selection

Articles were included if (1) they reported primary studies of diagnostic accuracy determined by comparing the results of the test under investigation with the results of a reference standard [1]; (2) they investigated a clinical population (no animals, fetuses, corpses, models, or phantoms); and (3) they used at least one measure of diagnostic accuracy such as sensitivity, specificity, likelihood ratios, predictive values, accuracy, and area under the receiver operator curve [27]. Systematic reviews, meta-analyses, letters, editorials, guidelines, statements, and comments were excluded. Not eligible were clinical trials and all studies of predictive accuracy. Firstly, two reviewers (A.S., A.T.) independently assessed the title, abstract, and keywords of all eligible articles to determine whether they met the inclusion criteria. Next, the full text of potentially eligible articles was evaluated by both reviewers. Disagreements were discussed and resolved in consensus meetings.

### Data extraction

The diagnostic accuracy studies finally included were evaluated by using the STARD checklist to assess the quality of reporting [2, 28]. The statement contains a list of 30 items [14]. Item 11 (rationale for choosing the reference standard (if alternatives exist)) was removed from the STARD checklist for this evaluation because the reviewers were not able to determine whether the item was not reported because no alternatives exist or because the authors did not mention it [29]. Thus, we here used a checklist of 29 items.

For this evaluation, two independent reviewers (A.S., A.T.) had to determine whether each item of the checklist was adequately described in the text. If the description was sufficient, they scored a point. The reviewers were not instructed to assess the likelihood of bias, but only the quality of reporting [30]. The two reviewers were not blinded to the source (year of publication, journal, authors) of the articles. Disagreements were discussed and resolved in consensus meetings. In case no consensus could be reached, a third reviewer (B.K., a physician with 8 years of experience in radiological research) made the final decision. The reviewers also noted the year of publication, the study design (cohort vs. case-control study), data collection (prospective vs. retrospective), and the citation rate of each article. The citation rate was calculated by dividing the number of citations of each article by August 31, 2021, as indicated by the citation index reported in the Web of Science (Thomson Reuters) by the number of months since publication date of the print version.

Although the STARD checklist is known to have good reproducibility [31, 32], we wanted to improve the accuracy of this assessment and make sure the two reviewers understood the items in the same way. Thus, there was a pilot testing. In this pilot phase, the two reviewers independently assessed four studies from 2014 and 2020 published in *Radiology* and *European Radiology* before evaluating the studies for this paper.

### Statistical analysis

For each article, the total number of items of the STARD statement that were described adequately was calculated (range, 0–29). The score can be interpreted as describing the quality of reporting—thus, the higher the specific score, the better the quality of reporting. Items 10, 12, 13, and 21 mention both the index test and the reference standard. To make sure equal weights to each of the items were applied, we counted the index test as 0.5 item and the reference standard as 0.5 item. The overall mean ± standard deviation (SD) and range of the total number of reported STARD items were calculated because the data were normally distributed. This was established by using the Shapiro-Wilk test. Articles were divided into two groups by publication date (year), study design, and method of data collection. Articles were also divided into two groups by median split for article citation rate. Student’s *t* test for independent samples was used to determine significant differences in the mean number of reported STARD items between the described groups.

For each item, the total number of articles that fulfilled the description of that item was counted separately (range, 0–114) and presented as percentages for 2015 and for 2019. Agreement between reviewers was determined as Cohen’s kappa with results classified as suggested by Landis and Koch (< 0.00, poor; 0.00–0.20, slight; 0.21–0.40, fair; 0.41–0.60, moderate; 0.61–0.80, substantial; and 0.81–1.00, almost perfect agreement between the reviewers) [33]. The median and interquartile range of the reading time of the two reviewers were calculated because the data were non-normally distributed, as shown by the Shapiro-Wilk test. *p* values smaller than 0.05 were considered significant. Statistical analysis was done by one reviewer (A.S.) under the supervision of a second reviewer (B.K.) by using IBM SPSS Statistics for Mac (Version 27.0.0.0).

## Results

### Search and selection

**Fig. 1:**
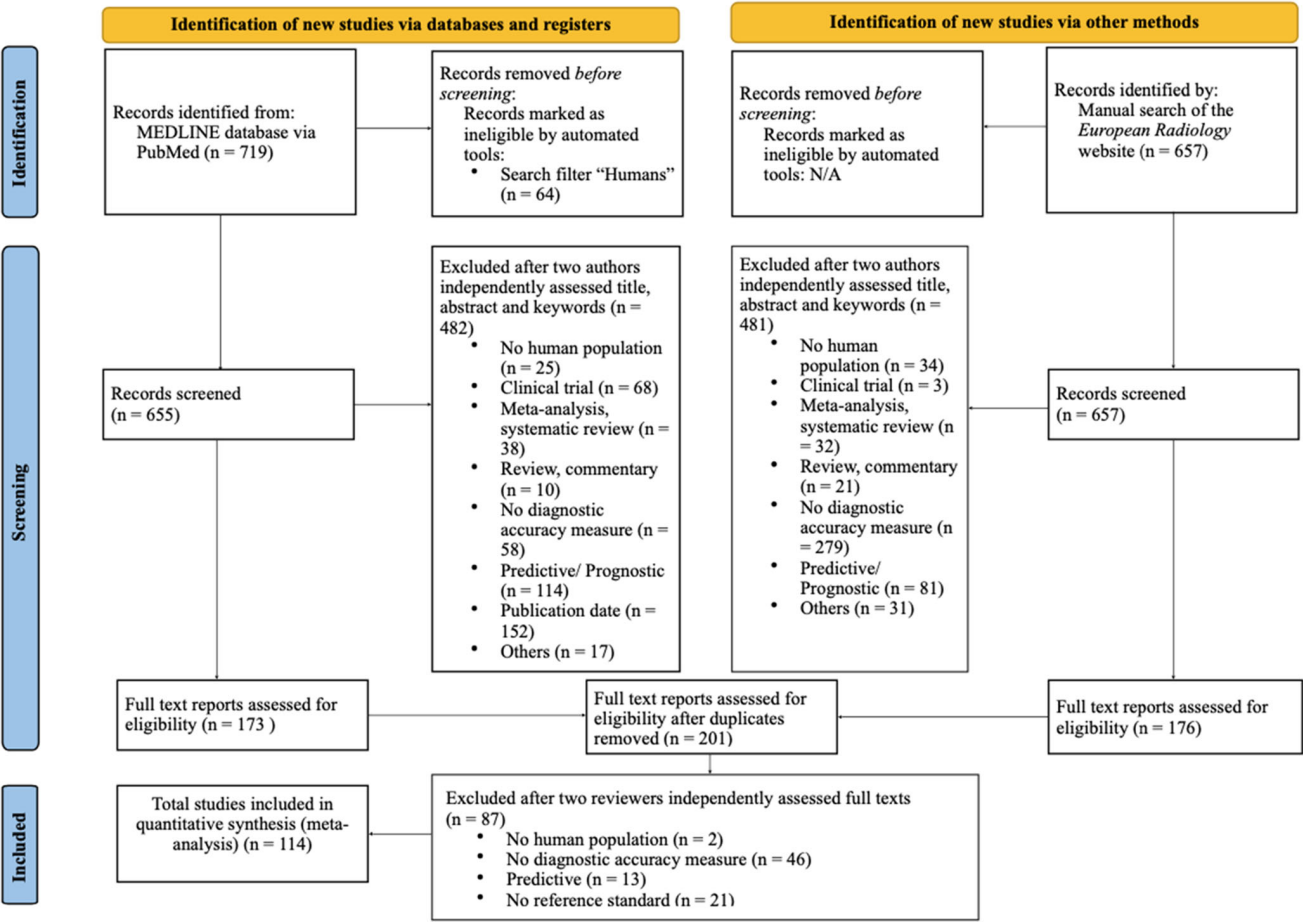
PRISMA 2020 flow diagram of selected diagnostic accuracy studies. N/A, not applicable

The search and selection process of diagnostic accuracy studies published in *European Radiology* in 2015 and 2019 is presented in the PRISMA flow diagram in Fig. 1. The database search on MEDLINE via PubMed identified 719 references. Another 657 studies were identified by manually searching the website of *European Radiology*. Based on the title, abstract, and keywords, two reviewers independently excluded 1027 articles for different reasons. The full texts of the remaining 201 studies were read by the two independent reviewers after removal of duplicates. Subsequently, 87 articles had to be excluded because they either used no measure of diagnostic accuracy (*n* = 46), the study determined predictive accuracies (*n* = 13), no human subjects were included (*n* = 2), no reference standard was used (*n* = 21), a model or algorithm was developed during the study process (*n* = 4), or a phantom was used (*n* = 1). Ultimately, we included 114 studies that met our selection criteria to investigate the quality of reporting. The median citation rate was 0.28 citation per month (IQR, 0.16–0.44). Details of the included articles by publication date are presented in Table 1.

**Table 1:** Characteristics of included studies

Study Characteristics	2015	2019
Total included studies	42	72
Study design		
Cohort	36	61
Case-control	6	11
Data collection		
Retrospective	19	39
Prospective	23	33
Citation rate (median split)		
Infrequently (≤ 0.28 citations/month)	25	32
Frequently (> 0.28 citations/month)	17	40

### Quality of reporting of diagnostic accuracy studies

**Fig. 2:**
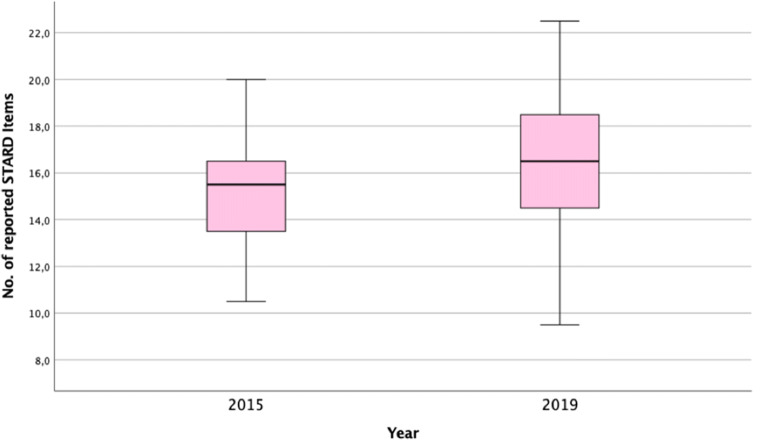
Median, interquartile ranges, and the range of adequately reported STARD items in 2015 (before STARD) and 2019 (STARD recommended). STARD, Standards for Reporting Diagnostic Accuracy; No., number

The overall mean total number of reported STARD items of all included 114 diagnostic accuracy studies was 15.9 ± 2.6 (54.8%) of 29 items (range, 9.5–22.5). The overall agreement of the reviewers in scoring the STARD items was 86.3%. Cohen’s kappa was 0.58 (95% CI, 0.49, 0.68), indicating moderate agreement between the reviewers. The median time needed for the evaluation per article was 19.5 min (IQR, 17.5–22). The complete list of included diagnostic accuracy studies with total STARD scores is provided in Appendix 1. The quality of reporting of diagnostic accuracy studies was significantly better in 2019 (mean ± SD, 16.3 ± 2.7) than in 2015 (mean ± SD, 15.1 ± 2.3; *p* < 0.02). No significant differences in the reported STARD items were identified in relation to study design (*p* = 0.13), data collection (*p* = 0.87), and citation rate (*p* = 0.09). Detailed results are provided in Table 2 and Fig. 2.

**Table 2:** Summary of performed subgroup analyses

Subgroup value	No. of STARD items reported, mean ± SD	*p*
Publication year		0.016
2015	15.1 ± 2.3	
2019	16.3 ± 2.7	
Study design		0.129
Cohort	16.1 ± 2.7	
Case-Control	15.0 ± 2.4	
Data collection		0.865
Retrospective	15.9 ± 2.4	
Prospective	15.9 ± 2.9	
Citation rate (median split)		0.094
Infrequently (< 0.28 citations/month)	15.5 ± 2.4	
Frequently (> 0.28 citations/month)	16.3 ± 2.8

### Quality of reporting of individual items

The quality of reporting in terms of the individual items of the STARD statement is presented in Table 3 both for all articles and separately for 2015 and 2019. Variation in the reporting quality across these items is very broad (0.9–100%). There are several items that were poorly reported (< 20%) such as the study objectives and hypotheses (item 4), information on how indeterminate index test or reference standard results were handled (item 15), the intended sample size and how it was determined (item 18), the cross tabulation of the index test results by the results of the reference standard (item 23), and any adverse events from performing the index test or the reference standard (item 25). Item 28 (registration number and name of registry) was only mentioned by one article of the 114 included diagnostic accuracy studies.

**Table 3:** Quality of reporting of the individual items of the STARD statement

Section and item no.	Item description	All articles	2015	2019
		(*n* = 114)	(*n* = 42)	(*n* = 72)
Title or abstract				
1	Identification as a study of diagnostic accuracy using at least one measure of accuracy (such as sensitivity, specificity, predictive values, or AUC)	113 (99.12)	42 (100)	71 (98.61)
Abstract				
2	Structured summary of study design, methods, results, and conclusions (for specific guidance, see STARD for Abstracts)	114 (100)	42 (100)	72 (100)
Introduction				
3	Scientific and clinical background, including the intended use and clinical role of the index test	112 (98.25)	41 (97.62)	71 (98.61)
4	Study objectives and hypotheses	17 (14.91)	6 (14.29)	11 (15.28)
Methods				
5	Whether data collection was planned before the index test and reference standard were performed (prospective study) or after (retrospective study)	103 (90.35)	37 (88.10)	66 (91.67)
6	Eligibility criteria	68 (59.65)	26 (61.90)	42 (58.33)
7	On what basis potentially eligible participants were identified (such as symptoms, results from previous tests, inclusion in registry)	105 (92.11)	37 (88.10)	68 (94.44)
8	Where and when potentially eligible participants were identified (setting, location, and dates)	65 (57.02)	22 (52.38)	43 (59.72)
9	Whether participants formed a consecutive, random, or convenience series	58 (50.88)	19 (45.24)	39 (54.17)
10	(a) Index test, in sufficient detail to allow replication	112 (98.25)	41 (97.62)	71 (98.61)
	(b) Reference standard, in sufficient detail to allow replication	51 (44.74)	19 (45.24)	32 (44.44)
12	(a) Definition of and rationale for test positivity cut-offs or result categories of the index test, distinguishing pre-specified from exploratory	54 (47.37)	19 (45.24)	35 (48.61)
	(b) Definition of and rationale for test positivity cut-offs or result categories of the reference standard, distinguishing pre-specified from exploratory	38 (33.33)	16 (38.10)	22 (30.56)
13	(a) Whether clinical information and reference standard results were available to the performers/readers of the index test	82 (71.93)	31 (73.81)	51 (70.83)
	(b) Whether clinical information and index test results were available to the assessors of the reference standard	28 (24.56)	9 (21.43)	19 (26.39)
14	Methods for estimating or comparing measures of diagnostic accuracy	55 (48.25)	12 (28.57)	43 (59.72)
15	How indeterminate index test or reference standard results were handled	11 (9.65)	7 (16.67)	4 (5.56)
16	How missing data on the index test and reference standard were handled	30 (26.32)	12 (28.57)	18 (25.00)
17	Any analyses of variability in diagnostic accuracy, distinguishing pre-specified from exploratory	69 (60.53)	21 (50.00)	48 (66.67)
18	Intended sample size and how it was determined	6 (5.26)	3 (7.14)	3 (4.17)
Results				
19	Flow of participants, using a diagram	45 (39.47)	9 (21.43)	36 (50.00)
20	Baseline demographic and clinical characteristics of participants	67 (58.77)	23 (54.76)	44 (61.11)
21	(a) Distribution of severity of disease in those with the target condition	101 (88.60)	39 (92.86)	62 (86.11)
	(b) Distribution of alternative diagnoses in those without the target condition	80 (70.18)	32 (76.19)	48 (66.67)
22	Time interval and any clinical interventions between index test and reference standard	62 (54.39)	23 (54.76)	39 (54.17)
23	Cross tabulation of the index test results (or their distribution) by the results of the reference standard	18 (15.79)	4 (9.52)	14 (19.44)
24	Estimates of diagnostic accuracy and their precision (such as 95% confidence intervals)	68 (59.65)	21 (50.00)	47 (65.28)
25	Any adverse events from performing the index test or the reference standard	13 (11.40)	5 (11.90)	8 (11.11)
Discussion				
26	Study limitations, including sources of potential bias, statistical uncertainty, and generalizability	91 (79.82)	35 (83.33)	56 (77.78)
27	Implications for practice, including the intended use and clinical role of the index test	109 (95.61)	38 (90.48)	71 (98.61)
Other information				
28	Registration number and name of registry	1 (0.88)	1 (2.38)	0 (0)
29	Where the full study protocol can be accessed	25 (21.93)	4 (9.52)	21 (29.17)
30	Sources of funding and other support; role of funders	114 (100)	42 (100)	72 (100)

In contrast, the following two items were adequately described by all studies including a structured summary of the study design, methods, results, and conclusions (item 2) and the sources of funding and other support (item 30). Frequently reported items (> 80%) were item 1 (identification as a study of diagnostic accuracy using at least one measure of accuracy), item 3 (scientific and clinical background), item 5 (whether data collection was planned before or after the index test and reference standard were performed), item 7 (on what basis potentially eligible participants were identified), item 10a (index test, in sufficient detail to allow replication), item 21a (distribution of severity of disease in those with the target condition), and item 27 (implications for practice).

As apparent from Table 3, there is a difference between the quality of reporting of the individual items between the years 2015 and 2019. Most of the items are reported more often in 2019 than in 2015. Several differences deserve special mention: methods for estimating or comparing measures of diagnostic accuracy (item 14, 28.6 to 59.7%), the use of a flow diagram (item 19, 21.4 to 50.0%), a cross tabulation of the index test results by the results of the reference standard (item 23, 9.5 to 19.4%), and the access to the full study protocol (item 29, 9.5 to 29.2%). The adherence to items related to the index test was generally better than the adherence to items concerning the reference standard (items 10, 12, 13).

## Discussion

The diagnostic accuracy of studies published in *European Radiology* slightly improved from 2015 to 2019. No correlation between the adherence to the STARD statement and the study design, the method of data collection, or the citation rate was found. Authors pay more attention to the description of the index test than that of the reference standard.

When we compare our results to other studies that have investigated the quality of reporting of diagnostic accuracy studies, the mean number of reported STARD items is higher (55%, 15.9/29) than identified recently by Hogan et al in 2020 (45%, 15.44/34). For items with subcomponents, they scored each subcomponent as an individual item, explaining the higher maximum possible score of 34 [23]. Similar to our results, the adherence to STARD was independent of the article citation rate [23]. Also, Choi et al found no significant correlation between the total STARD score and the total number of citations. Note though that Choi et al did not use the citation rate but the total number of citations [16]. Hong et al identified nearly the same mean number of reported STARD items in imaging journals in 2018 (55%, 16.6/30) as found in our analysis. No significant difference regarding the study design was found, which corresponds to our results as well [18]. In contrast, Walther et al identified a higher adherence to STARD (69%, 14.4/21) in 2014 using the original STARD checklist published in 2003 and focusing on studies of coronary CT angiography [22]. But they also found a significant improvement over time (2003–2011) with a 0.3-point increase in the total STARD score per year [22]. Smidt et al identified a better quality of reporting in cohort studies than in case-control studies in 2006, but pointed out that, in general, case-control studies are able to fulfill all individual items [15]. We also found a higher mean number of reported STARD items in cohort studies, but the difference was not significant.

Looking at the quality of reporting by item, we found a broad variation (0.9–100%), as did Michelessi et al in 106 studies focusing on diagnostic accuracy research in glaucoma in 2017 (0–100%) [17]. In agreement with the results for individual items reported by Choi et al, Hong et al, and Hogan et al, we identified item 18 (intended sample size and how it was determined) and item 25 (any adverse events from performing the index test or the reference standard) as consistently poorly reported (< 20%) [16, 18, 23]. This is especially worth mentioning as these items can be reported in a few sentences.

Our study has several limitations. Firstly, the validated search strategy we used to identify all relevant diagnostic accuracy studies on MEDLINE via PubMed that met our inclusion criteria has a sensitivity of 80.0% and a specificity of 97.3% [26]. It is thus likely that we did not find all eligible diagnostic accuracy studies. While we tried to reduce the number of missed studies by additionally searching the website of *European Radiology* manually, it is still possible that some potentially eligible studies are not included. Secondly, we changed the original STARD checklist by excluding item 11 because a meaningful decision about adherence was not possible. Thirdly, we only investigated diagnostic accuracy studies published in *European Radiology.* The last two points may impair the generalizability of our study, but we are still able to draw a conclusion about the quality of reporting of diagnostic accuracy studies in *European Radiology* for 2015 and 2019. Fourthly, although the STARD statement comes along with detailed explanations and is highly elaborated [2], there remains some subjectivity in scoring the items. To reduce the observer bias, we had a pilot testing before the present study was conducted, and the two reviewers assessed each study independently. Additionally, if no consensus could be reached, a third reviewer helped to make the final decision. Finally, by assigning either 0 points (insufficient description) or 1 point (sufficient description) per item, our scoring system was less fine grained than the approach used by Zafar et al, who scored each item as completely reported (score = 2), partly reported (score = 1), or not reported (score = 0) [34]. Our scoring method could have a negative effect on items that need to be described in more detail such as item 6 (eligibility criteria) or item 10b (reference standard, in sufficient detail to allow replication).

In conclusion, the quality of reporting of diagnostic accuracy studies has improved rather moderately since *European Radiology* started encouraging its authors to follow the STARD guidelines. Authors and reviewers should pay more attention to adherence to the various items of the STARD checklist to avoid any kind of bias. Journal editors can make a contribution to improved reporting by recommending the use of the STARD statement or by making it mandatory for articles to be accepted for publication.

## Supplementary information

ESM 1 (PDF 208 kb)

## Abbreviations

CI: Confidence interval
IQR: Interquartile range
PRISMA: Preferred Reporting Items for Systematic Reviews and Meta-Analyses
PROSPERO: International Prospective Register of Systematic Reviews
SD: Standard deviation
STARD: Standards for Reporting Diagnostic Accuracy

## Declarations

### Guarantor

The scientific guarantor of this publication is Prof. Dr. Marc Dewey.

### Statistics and biometry

No complex statistical methods were necessary for this paper.

### Methodology

• retrospective

• not applicable

• performed at one institution
