# Supplementary material for: Has the STARD statement improved the quality of reporting of diagnostic accuracy studies published in European Radiology?
Source: Eur Radiol. 2022 Jul 30;33(1):97–105. doi: 10.1007/s00330-022-09008-7 (PMC9362582; doi:10.1007/s00330-022-09008-7)
Supplement: Supplementary file 1 — (PDF 208 kb) [file 330_2022_9008_MOESM1_ESM.pdf]

Table A1. Complete list of all included studies with overall STARD scores

No.	Year	Author	Total STARD Score	DOI
1	2015	Lubina	13.5	10.1007/s00330-014-3521-2
2	2015	Saito	15.5	10.1007/s00330-014-3373-9
3	2015	Dornberger	19.5	10.1007/s00330-015-3776-2
4	2015	Wu	16	10.1007/s00330-015-3774-4
5	2015	Kim	14.5	10.1007/s00330-015-3762-8
6	2015	Zhao	16.5	10.1007/s00330-015-3592-8
7	2015	Litjens	17.5	10.1007/s00330-015-3743-y
8	2015	Atema	15.5	10.1007/s00330-015-3648-9
9	2015	Kaiser	14.5	10.1007/s00330-014-3580-4
10	2015	Zhao	12.5	10.1007/s00330-015-3634-2
11	2015	Zhao	14	10.1007/s00330-014-3465-6
12	2015	Lohmann	10.5	10.1007/s00330-015-3691-6
13	2015	Cheng	15.5	10.1007/s00330-015-3638-y
14	2015	Alakhras	10.5	10.1007/s00330-014-3409-1
15	2015	Zhang	11	10.1007/s00330-014-3407-3
16	2015	Nakajo	17.5	10.1007/s00330-015-3787-z
17	2015	Yu	18.5	10.1007/s00330-015-3615-5
18	2015	Walter	16	10.1007/s00330-014-3520-3
19	2015	Grgurevic	15.5	10.1007/s00330-015-3728-x
20	2015	Yan	14	10.1007/s00330-014-3432-2
21	2015	Low	13	10.1007/s00330-015-3723-2
22	2015	Breitenseher	11	10.1007/s00330-015-3613-7
23	2015	Bandirali	16	10.1007/s00330-015-3699-y
24	2015	Fischer	15.5	10.1007/s00330-015-3732-1
25	2015	Kato	12	10.1007/s00330-015-3755-7
26	2015	Manganaro	17	10.1007/s00330-015-3766-4
27	2015	Petrillo	15	10.1007/s00330-014-3581-3
28	2015	Zheng	15.5	10.1007/s00330-014-3519-9
29	2015	Brisse	18.5	10.1007/s00330-014-3514-1

Table A1. Complete list of all included studies with overall STARD scores

30	2015	Shin	15.5	10.1007/s00330-014-3399-z
31	2015	Kobayashi	16.5	10.1007/s00330-015-3725-0
32	2015	Kauv	20	10.1007/s00330-015-3795-z
33	2015	Liu	13	10.1007/s00330-015-3611-9
34	2015	Omoumi	15	10.1007/s00330-014-3469-2
35	2015	Zhang	12	10.1007/s00330-014-3462-9
36	2015	Tagliafico	16.5	10.1007/s00330-015-3645-z
37	2015	Derlin	16.5	10.1007/s00330-014-3503-4
38	2015	Joo	17	10.1007/s00330-015-3686-3
39	2015	Steggerda	14.5	10.1007/s00330-015-3665-8
40	2015	Eom	16	10.1007/s00330-014-3547-5
41	2015	Ringl	17	10.1007/s00330-015-3598-2
42	2015	Inchingolo	13.5	10.1007/s00330-014-3500-7
43	2019	Dyrberg	22.5	10.1007/s00330-018-5682-x
44	2019	Li	19	10.1007/s00330-018-5860-x
45	2019	Zirpoli	13.5	10.1007/s00330-019-06042-w
46	2019	Langenbach	17	10.1007/s00330-019-06297-3
47	2019	Alqahtani	9.5	10.1007/s00330-019-06250-4
48	2019	Bae	20	10.1007/s00330-019-06272-y
49	2019	Lee	16.5	10.1007/s00330-018-5716-4
50	2019	Rafailidis	17	10.1007/s00330-018-5773-8
51	2019	Gillet	15.5	10.1007/s00330-018-5717-3
52	2019	Jeong	18	10.1007/s00330-018-5772-9
53	2019	Onoue	16	10.1007/s00330-019-06107-w
54	2019	Chae	15.5	10.1007/s00330-018-5886-0
55	2019	Krishna	15	10.1007/s00330-018-5664-z
56	2019	Pourvaziri	18.5	10.1007/s00330-019-06224-6
57	2019	Goto	14.5	10.1007/s00330-018-5643-4
58	2019	Wang	12	10.1007/s00330-018-5977-y
59	2019	Pumberger	20.5	10.1007/s00330-018-5963-4

Table A1. Complete list of all included studies with overall STARD scores

60	2019	Xu	12.5	10.1007/s00330-019-06311-8
61	2019	Weng	15	10.1007/s00330-019-06339-w
62	2019	Tu	13.5	10.1007/s00330-018-5839-7
63	2019	Zheng	15.5	10.1007/s00330-018-5676-8
64	2019	Gersing	13	10.1007/s00330-018-5450-y
65	2019	Engel	13.5	10.1007/s00330-018-5769-4
66	2019	Lobbes	14.5	10.1007/s00330-019-06215-7
67	2019	Kocak	15.5	10.1007/s00330-018-5652-3
68	2019	Markhardt	13	10.1007/s00330-019-06143-6
69	2019	Ruan	15.5	10.1007/s00330-018-5992-z
70	2019	Lee	14	10.1007/s00330-018-5893-1
71	2019	Dai	18.5	10.1007/s00330-018-5777-4
72	2019	Jia	12	10.1007/s00330-019-06111-0
73	2019	Abdullayev	19	10.1007/s00330-019-06233-5
74	2019	Wei	14	10.1007/s00330-018-5638-1
75	2019	Mürtz	14.5	10.1007/s00330-019-06192-x
76	2019	Zhang	21.5	10.1007/s00330-018-5848-6
77	2019	Grgurevic	15	10.1007/s00330-018-5831-2
78	2019	Barchetti	16.5	10.1007/s00330-019-06117-8
79	2019	Johnston	21	10.1007/s00330-018-5813-4
80	2019	Lukas	12.5	10.1007/s00330-018-5899-8
81	2019	Seo	19.5	10.1007/s00330-018-5557-1
82	2019	Uhlig	14.5	10.1007/s00330-018-5854-8
83	2019	Keller	15.5	10.1007/s00330-018-5614-9
84	2019	Bae	19	10.1007/s00330-019-06123-w
85	2019	Joo	13	10.1007/s00330-018-5727-1
86	2019	Liang	19	10.1007/s00330-018-5929-6
87	2019	Xiang	16	10.1007/s00330-019-06274-w
88	2019	Diekhoff	19	10.1007/s00330-018-5568-y
89	2019	Hryniewiecki	9.5	10.1007/s00330-018-5965-2

Table A1. Complete list of all included studies with overall STARD scores

90	2019	Neumann	18	10.1007/s00330-018-5623-8
91	2019	Botsikas	16.5	10.1007/s00330-018-5720-8
92	2019	Jhaveri	15	10.1007/s00330-018-5619-4
93	2019	in´t Veld	17.5	10.1007/s00330-019-06234-4
94	2019	Avanesov	17	10.1007/s00330-019-06279-5
95	2019	Girard	17.5	10.1007/s00330-018-5959-0
96	2019	Pellerin	19	10.1007/s00330-019-06173-0
97	2019	Bise	17	10.1007/s00330-018-5784-5
98	2019	Larbi	16.5	10.1007/s00330-018-5796-1
99	2019	Zhang	16	10.1007/s00330-018-5633-6
100	2019	Austein	14.5	10.1007/s00330-019-06252-2
101	2019	Byun	18	10.1007/s00330-018-5905-1
102	2019	Caglic	19	10.1007/s00330-019-06070-6
103	2019	Martin	18	10.1007/s00330-018-5844-x
104	2019	Plodeck	15.5	10.1007/s00330-018-5589-6
105	2019	Seif Amir Hosseini	18	10.1007/s00330-019-06284-8
106	2019	Bonekamp	17	10.1007/s00330-018-5751-1
107	2019	Canellas	18.5	10.1007/s00330-019-06113-y
108	2019	Ge	13.5	10.1007/s00330-019-06226-4
109	2019	De Santis	21.5	10.1007/s00330-019-06032-y
110	2019	Bakhshayeshkaram	17	10.1007/s00330-019-06106-x
111	2019	Barat	18.5	10.1007/s00330-018-5734-2
112	2019	Hong	18.5	10.1007/s00330-018-5817-0
113	2019	Tu	15	10.1007/s00330-018-5778-3
114	2019	Gollub	19	10.1007/s00330-018-5719-1

Untertitel
